# Gliclazide Enhances Exercise Performance and Recovery of Muscle Strength in Healthy Trained Individuals: A Randomized Controlled Trial

**DOI:** 10.1002/ejsc.70155

**Published:** 2026-03-12

**Authors:** Jocelito Bijoldo Martins, Thiago Dipp, Beatriz D. Schaan, Alexandre Machado Lehnen

**Affiliations:** ^1^ Instituto de Cardiologia do Rio Grande do Sul/Fundação Universitária de Cardiologia Porto Alegre RS Brazil; ^2^ Programa de Pos‐Graduação em Saúde Coletiva Universidade do Vale do Rio dos Sinos São Leopoldo Rio Grande do Sul Brazil; ^3^ Hospital de Clínicas de Porto Alegre Porto Alegre RS Brazil; ^4^ Universidade Federal do Rio Grande do Sul Porto Alegre RS Brazil

**Keywords:** doping in sports, glucose, insulin, muscle recovery, muscle strength

## Abstract

To examine the acute effect of gliclazide on exercise performance and recovery of muscle strength in healthy participants. We conducted a randomized, double‐blind, placebo‐controlled crossover clinical trial in 44 strength‐trained men. They were allocated to gliclazide modified release (MR) (90 mg, 8h before exercise sessions) or placebo, undergo three consecutive sessions of strength exercise (four sets, 80% of one‐repetition maximum [1‐RM] of bench press and free squat exercise). We evaluated total volume‐load (VL) (#repetitions x 80%1‐RM), range of motion (ROM), insulin and glucose levels, creatine kinase MM (CK‐MM), lactate dehydrogenase (LDH), interleukin‐6 (IL‐6) and tumor necrosis factor alpha (TNF‐α), hemodynamic parameters, perceived muscle soreness and recovery scores. Gliclazide enhanced strength exercise performance with improvements in total VL (bench press 23.3%, *p* < 0.001; squat 23.2%, *p* < 0.001), and improved muscle recovery 24–48h post‐exercise: ROM (shoulder 1.1%, *p* < 0.001; knee 1.6%, *p* = 0.004), CK‐MM (−13.2%, *p* < 0.001), LDH (−12.8%, *p* < 0.001), TNF‐α (−17.4%, *p* < 0.001), IL‐6 (−5.3%, *p* < 0.001), muscle soreness (−17.7%, *p* < 0.001) and recovery scores (32.5%, *p* = 0.001). However, hypoglycemia events were observed in 3 participants in the gliclazide group. In conclusion, Gliclazide MR 90 mg, 8h before strength exercise, produced ergogenic effects (exercise performance and muscle recovery), although hypoglycemia was observed in 7% of subjects. **Registration**: “www.clinicaltrials.gov”, “NCT04443777” (Primary Completion: 01/08/2020; Study Completion: 31/10/2023)

## Highlights

- Gliclazide MR acutely increases strength and muscle recovery.
- Markers decrease: CK‐MM, LDH, TNF‐α, IL‐6; 1‐RM and ROM recover.
- In athletes, Gliclazide MR may cause adverse events, notably hypoglycaemia.
- Off‐label use must balance ergogenic benefit against safety risks.

## 1 Introduction

Sulfonylureas are a class of drugs that has been used in the treatment of type 2 diabetes mellitus (T2DM) (Manu et al. 2021). They act by binding to a specific receptor for sulfonylureas (SUR) in pancreatic *β*‐cells, blocking ATP‐dependent potassium channels (K^+^‐ATP) and stimulating the secretion of insulin (Keitel 2013). The World Health Organization (WHO) list of medications includes gliclazide as the safest and most effective drug in the sulfonylurea class and is indicated for the treatment of T2DM (Draznin 2022). Due to its pharmacodynamic properties, gliclazide is highly selective and binds to receptors on pancreatic *β*‐cells (SUR1) and at the same time shows low affinity for receptors on cardiac muscle (SUR2A) and vascular muscle cells (SUR2B) (Proks et al. 2002).

Numerous athletes use substances and/or methods for performance enhancement which is against the spirit of sports and may be harmful to their health. One of these substances is insulin, an anabolic hormone that can artificially increase muscle mass and enhance athletic performance. Because of its use for doping in sports, the World Anti‐Doping Agency (WADA) has put insulin in the list of prohibited substances (WADA 2023). Yet, there are reports of the use of sulfonylureas as an insulinotropic drug in amateur and professional athletes since they are not included in this list of doping agents.

Several hypotheses may be advanced regarding the potential ergogenic effects of gliclazide, a sulfonylurea: (i) sulfonylureas stimulate endogenous insulin secretion, thereby enhancing skeletal‐muscle glucose and amino‐acid uptake, which may improve substrate availability, tissue recovery, and performance (Flores‐Opazo et al. 2020; Richter et al. 2021); (ii) they can promote glycogen resynthesis and accelerate post‐exercise recovery across repeated sessions (Mikines et al. 1988; Roberts et al. 2013); and (iii) clinical data indicate that gliclazide treatment reduces plasma IL‐6 with a concomitant trend toward lower TNF‐α, attenuates oxidative stress, suppresses NF‐κB activation (Jahan and Choudhary 2021), and promotes macrophage polarization—collectively supporting an anti‐inflammatory effect (Drzewoski and Zurawska‐Klis 2006). Consistent with these mechanisms, a pilot study from our group found that a single pre‐exercise dose of modified‐release gliclazide (60 mg) did not directly enhance acute strength performance, although it appeared to facilitate recovery in subsequent sessions (Martins et al. 2024).

Despite these hypotheses, the hypothetical effect of gliclazide as a potential ergogenic substance has not yet been tested, neither as an acute nor as a chronic response to exercise. The closest approach involved studies that acutely evaluated healthy participants taking gliclazide for its bioequivalence (Pop et al. 2019; Rojanasthien et al. 2012) and pharmacokinetic and pharmacodynamic properties (Kobayashi et al. 1984; Samad et al. 2011), studies that investigated gliclazide metabolism and polymorphic hepatic enzymes involved (Chow et al. 2019; Shao et al. 2010), and one study that evaluated relatively prolonged use (7 days) of gliclazide (Ling et al. 2006). However, none of them examined athletic performance or post‐exercise muscle recovery as an outcome.

Hence, the objective of the present study was to examine the acute effect of gliclazide MR on strength performance and markers of muscle recovery following three strength exercise sessions in healthy trained participants. We hypothesize that gliclazide MR 90 mg once daily enhances strength performance and improves (accelerates) muscle recovery.

## 2 Methods

This study is a randomized, double‐blind, placebo‐controlled crossover clinical trial. The study was approved by the research ethics committee at Instituto de Cardiologia do Rio Grande do Sul/Fundação Universitária de Cardiologia (protocol number 3,771,137). It followed the principles of the Declaration of Helsinki, is registered in www.clinicaltrials.gov (ID NCT04443777, Primary Completion: 01/08/2020; Study Completion: 31/10/2023) (Cuschieri 2019) and is reported following the CONSORT guidelines.

### 2.1 Study Participants

The sample size was estimated based on a pilot crossover randomized clinical trial of our research group involving 10 healthy strength trained participants that evaluated strength performance (total volume‐load [VL] in kg) as the primary outcome (Martins et al. 2024). For the expected effect size of the study (*d* = 0.25), a power of 90%, a significance level of 5% and an additional 20% of sample loss, we calculated it was necessary a sample of 44 participants. We used GPower v3.1 for sample calculation.

The inclusion criteria were healthy men aged 20–35 years, minimum of two years of resistance training (average training frequency of three times per week), corresponding to a “trained/highly trained” classification based on training history and exposure, rather than competitive level, according to McKay et al. (2022), and uninterrupted training in the preceding six months. We excluded those who self‐reported use of anabolic androgenic steroids or exogenous insulin in the preceding 24 months, any musculoskeletal injuries during data collection, and acute or chronic use of drugs that could affect strength performance during exercise sessions. The volunteers were strongly advised to avoid drinking alcohol for 72 h before the study exercise sessions.

Also, the participants were asked to follow a personalized diet plan prescribed by a nutritionist during the study. The diet plan consisted of an intake of 1.4 g protein/kg (∼20% of the total energy value), 4.2 g carbohydrate/kg (∼55%) and 0.85 g lipid/kg (∼25%) (Burke 2021). To prevent potential hypoglycemic events after exercise, participants were offered a snack before and after the sessions including a cheese and turkey breast sandwich made with whole grain bread and a cup of fruit yogurt consisting of 0.5 g/kg proteins and 0.88 g/kg carbohydrates (Burke 2021).

### 2.2 Description of Study Visits

**FIGURE 1:**
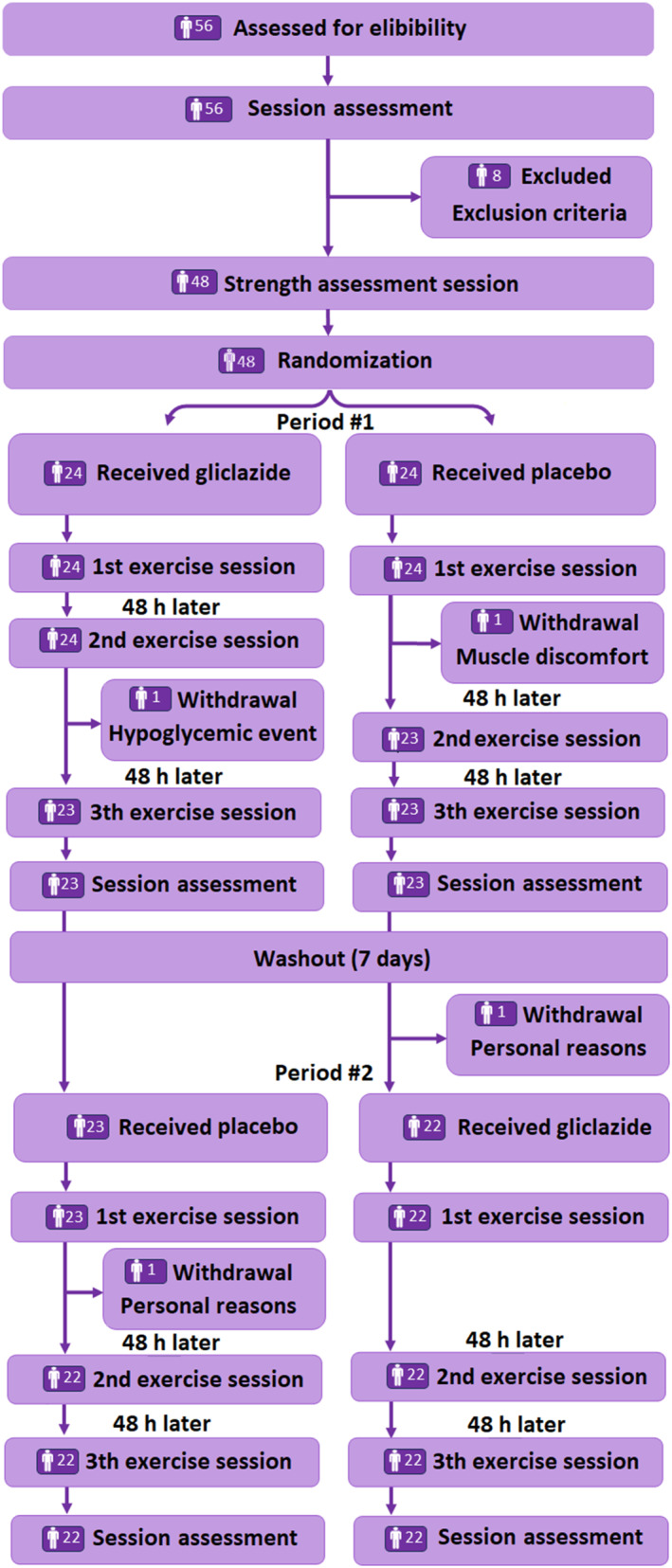
Flow chart consort of the study.

All participants completed a total of 12 visits at the study site. On visits 1 and 2, they underwent general assessment and maximal dynamic strength tests. They performed three exercise sessions on visits 3, 4, and 5, with a 48‐h interval between them. Visits 6 and 7 were dedicated to blood collection and the assessment of muscle recovery (24 and 48 h after the last exercise session). After a one‐week washout, participants completed three additional strength‐training sessions and returned for two subsequent visits at 24 and 48 h for blood sampling and muscle‐recovery assessments (Figure 1). All volunteers were instructed not to engage in any other type of exercise throughout the study.

On visit 1 (evaluation session), the participants were first explained the study procedures, risks and benefits and those who agreed to participate signed an informed consent form. They underwent a medical interview (medical, family and exercise information were collected). Resting heart rate (HR) (Polar RS300) and blood pressure (BP) (Omron 7122 digital sphygmomanometer) were measured. They were also instructed to fast for 8 h before venous blood collection (10 mL) for plasma insulin and glucose determination. We also conducted an anthropometric evaluation. Body mass (Fillizola scale; precision of 0.05 kg) and height were measured. Skinfold thickness measurements were taken in the right hemibody (chest, mid‐axillary, triceps, subscapular, abdominal, supra‐iliac and mid‐thigh) in triplicate non‐consecutively with a scientific skinfold caliper (Cescorf; precision of 0.1 mm) to estimate body density from the Jackson and Pollock equation (Jackson and Pollock 1978). Body composition parameters, including absolute and relative fat and fat‐free mass, were evaluated using the Siri equation (Siri 1993). At the end of visit 1, the participants had an opportunity to get familiar with bench press and free squat exercises for the 1‐RM test. They were instructed to return 2 days later to perform this test.

On visit 2 (one‐repetition maximum test), the participants performed the 1‐RM test according to published procedures (Correa et al. 2013; Correa 2012). The resulting 1‐RM values were used to prescribe the loads for the strength‐training sessions (visits 3, 4, and 5).

In turn, on visits 3, 4 and 5 (strength exercise sessions), the participants completed three strength exercise sessions 48 h apart. Upon arrival at the study site, they rested for 10 min before HR and BP measurements and fasting blood collection (10 mL) were done. They were offered a snack before exercise. Rrange of motion (ROM) for shoulder horizontal abduction and knee flexion was assessed before warm‐up (on visit 3 only). HR and BP were measured and muscle soreness was rated using the visual analog scale (VAS) (Thong et al. 2018) pre‐exercise before warm‐up, between sets of exercise and at the end of the session (post‐exercise). Importantly, muscle soreness assessed during the exercise sessions reflects acute effort‐related muscle discomfort, commonly described as a burning or pressure sensation associated with metabolite accumulation, transient ischemia, and nociceptor activation during high‐intensity muscle contractions. Thus, VAS collected during the exercise sessions were used solely to characterize perceived discomfort during effort. In contrast, perceived muscle soreness and recovery scores assessed during the 24 and 48 h recovery period were considered indicators of post‐exercise recovery. Also, a 10‐mL sample of venous blood was collected immediately after exercise. Capillary blood glucose was measured using a meter (AccuChek glucometer) before and after the exercise sessions. The volunteers were offered a snack after the exercise session to prevent hypoglycemic events. In addition, they were also strongly advised not to engage in any form of physical exertion beyond that of the study and to follow the prescribed diet plan.

On visits 6 and 7 (assessment of muscle recovery), the muscle recovery assessments were carried out 24 and 48 h after the exercise session on visit 5. The participants were asked to rest for 10 min before measuring HR, BP, VAS and perceived recovery scores (PRS) (Laurent et al. 2011). They also underwent blood collection (10 mL sample), ROM assessment and 1‐RM test in a row. After visit 7, the volunteers underwent a washout period of 1 week. They were advised not to perform any physical exercise and keep a standard diet. On day 8 they returned to the study site for the period 2 of crossing over. They received new tablets and repeated the procedures of visits 3 to 5, totaling 12 visits per participant.

#### 2.2.1 Procedures and Assessments

For one‐repetition maximum test, the participants were asked to get familiar with exercise sets (48 h before the experimental session). Following a 5‐min warm‐up on a cycle ergometer, participants performed exercise‐specific rehearsal sets for the bench press and free squat at an initial workload of 15%–20% of body mass to warm up the target musculature and standardize movement velocity and range of motion. Thereafter, the maximal load (1‐RM) for the free squat and bench press (TUTECH Fitness, 3‐inch model) was determined as the greatest load successfully lifted within five attempts using full range of motion, with 5 min of rest between attempts. The intraclass correlation coefficients (ICCs) of one‐repetition maximum (1‐RM) tests for bench press and squat during the familiarization and pre‐session evaluation sessions were 0.96 and 0.97, respectively. At the end of this session the participants were given guidance for the next visits.

We used VL (volume‐load) as a parameter of strength performance. The absolute load lifted (80% of 1‐RM), total number of repetitions up to failure for each type of exercise (bench press and free squat) and the number of sets (4 sets) were recorded on Visits 3, 4 and 5. VL was calculated as the load lifted (kg) x total number of repetitions per set x number of sets for each type of exercise (Correa 2012).

We assessed ROM using an extensible goniometer (Gonnext, Brazil). Horizontal abduction ROM of the shoulder was measured with the participants lying prone on the examination table, their shoulder positioned at 90 degrees of abduction keeping the elbow extended and palm down. The axis of the goniometer was positioned on the superior‐lateral aspect of the acromion. The stationary arm was positioned over the humerus. The movable arm was parallel to the humerus using the radial styloid process for reference following the horizontal abduction motion (Norkin and White 2016). Knee flexion ROM was measured with the participants lying prone with their knees extended. The axis of the goniometer was positioned on the lateral epicondyle of the femur. The stationary arm was on the lateral midline of the femur using the greater trochanter for reference. The movable arm was in the lateral midline of the fibula using the lateral malleolus for reference (Norkin and White 2016). ROM was measured in degrees from the range of the device's stationary and moving arms.

Blood samples were collected through vacuum venipuncture in the antecubital area of the arm using an aseptic technique. Blood tubes were centrifuged and the supernatant (plasma or serum) was aliquoted and frozen at −80°C for subsequent analysis. Accordingly, the analysis of blood markers was conducted as described below.

Plasma insulin concentration was determined through a chemiluminescent immunoassay (COBAS 6000, Roche Brazil) according to the manufacturer's guidelines (Insulin Elecsys Reagent, Roche Diagnostics, Germany).

Creatine kinase MM (muscle‐muscle) isoform (CK‐MM) and lactate dehydrogenase (LDH) are indirect markers of exercise‐induced muscle damage (Romero‐Moraleda et al. 2019). The enzymatic activity of CK‐MM and LDH levels were determined in duplicate using a colorimetric enzymatic assay (Roche, Analysis 7096) according to the manufacturer's guidelines (Creatina Kit ECPK‐100, 340 nm and Lactato Desidrogenase Kit LDH, 450 nm, LEAC, Brazil). CK‐MM is most abundant in skeletal muscle (95% of total CK) and therefore is the most specific marker of exercise‐induced muscle damage (Cecil 2012). To eliminate inter‐assay variance, all samples were analyzed within the same assay batch (intra‐assay variances ≤ 5.9%). ICCs were 0.96 for CK‐MM and 0.94 for LDH.

As the muscle recovery process involves mechanisms associated with the inflammatory state, we evaluated interleukin‐6 (IL‐6) and tumor necrosis factor alpha (TNF‐α). Thus, plasma IL‐6 and TNF‐α levels were assayed in duplicate using ELISA (MB‐580 model, Heales Heales) according to the manufacturer's guidelines (R&D Systems, Minneapolis, US).

#### 2.2.2 Intervention With Gliclazide and Placebo

The participants were randomly allocated to receive gliclazide 90 mg as a modified‐release tablet (Diamicron MR) or placebo (starch) and then crossed over. Randomization of the study intervention sequence was carried out using a computer program (www.randomizer.org). An external researcher managed the randomized list and the tablets (gliclazide or placebo) for the study. We used a potentially safe gliclazide dose of 75% of the maximum clinically recommended dose (120 mg) (Kobayashi et al. 1984; Samad et al. 2011).

A blinded investigator provided the calculated number of tablets required for each volunteer after their initial assessment (see Visit 2 section). Tablets containing gliclazide or placebo were matched by color, size, flavor and smell. The participants were instructed to take one tablet orally 8 h before each exercise session (see Visits 3, 4 and 5 section). On the days between visits (interval of 48 h), the tablets were to be taken with breakfast. The timing of tablet administration on the day of the study session was set so that peak plasma concentration of the drug would coincide with exercise, as gliclazide MR have linear pharmacokinetic properties with increasing plasma levels within 6 h after administration, reaching a plateau after 12 h and lasting effect up to 24 h (Kim et al. 2003).

We allowed a washout period of 1 week when we switched between the two periods of the study (gliclazide or placebo before exercise) as recommended due to gliclazide's half‐life of 16 h (metabolized by the liver) and clearance of 0.9 L/h (Schernthaner 2003).

#### 2.2.3 Strength Exercise Protocol

The strength exercise protocol started with dynamic warm‐up involving sequential bench press and squat exercises with individualized loads of 50% of 1‐RM. The participants were allowed a 2‐min rest before the main part of the session: four sets of exercise at 80% of 1‐RM. An electronic metronome was used to control each repetition (2:2) until they were no longer able to complete the repetition cycle (concentric failure). The number of complete repetitions was counted. They were asked to perform a set of bench press followed by free squat with no rest between exercises and then a 3‐min rest was allowed.

### 2.3 Statistical Analysis

The data are presented as mean ± standard deviation (SD). We used the Shapiro‐Wilk test to assess the assumption of normality and the Levene test to assess homogeneity of variance. When normality was not met, the data were log‐transformed. We performed paired Student's *t*‐test for exercise type and set to compare total VL of exercise sessions between the gliclazide and placebo groups. We used repeated measures two‐factor ANOVA or Generalized Estimating Equations (GEE), with Bonferroni post‐hoc tests to compare variables pre‐exercise, during the three exercise sessions and in the recovery period (24 and 48 h post‐exercise) between the gliclazide and placebo groups. When the condition of sphericity was not met, the Greenhouse‐Geisser correction was used to adjust for the significance level of the test. Cohen's d was used to determine the effect size and classified as non‐relevant (< 0.20), small (0.20–0.49), medium (0.50 and 0.79), and large or consistent (> 0.80). All data were analyzed using SPSS v23.0 at an alpha level of 5% (*p* < 0.05).

## 3 Results

A total of 56 volunteers were recruited at Instituto de Cardiologia do Rio Grande do Sul/Fundação Universitária de Cardiologia and eligible to participate in the study from August 01, 2020 and study completion October 10, 2023 (including period of the COVID‐19 pandemic). Of 56 volunteers, eight were excluded and 48 were included in the study. Four individuals did not complete the study protocol—one had a hypoglycemic event, two experienced muscle discomfort, and one gave up because of personal reasons. Our final sample comprised 44 participants, which matched the estimated sample size. Table 1 shows physical and clinical characteristics of the participants.

**TABLE 1:** Physical and physiological characteristics of the study sample (*n* = 44).

Variables	Mean ± SD
Age (years)	28.4 ± 3.24
Total body mass (kg)	83.9 ± 8.9
Height (cm)	178.0 ± 4.8
Relative fat mass (%)	22.8 ± 3.1
Absolute fat mass (kg)	19.3 ± 4.2
Relative fat‐free mass (%)	77.2 ± 3.1
Absolute fat‐free mass (kg)	64.7 ± 5.8
Sum of seven skinfolds (mm)	85.0 ± 19.5
Strength training experience (years)	3.8 ± 1.2
1‐RM bench press (kg)	100.5 ± 17.2
1‐RM squat (kg)	113.1 ± 17.9
Fasting blood glucose (mg/dL)	92.7 ± 7.3
Fasting insulin (μU/mL)	5.6 ± 1.0
HOMA‐IR	1.3 ± 0.1
HOMA II (%)	0.74 ± 0.2
Insulin sensitivity	135.9 ± 5.9
β‐cell function	72.4 ± 3.6

*Note:* HOMA‐IR was calculated using the homeostatic model assessment (HOMA) II calculator; insulin sensitivity and *β*‐cell function were determined using an application designed by University of Oxford (Wallace et al. 2004).

### 3.1 Strength Performance

**FIGURE 2:**
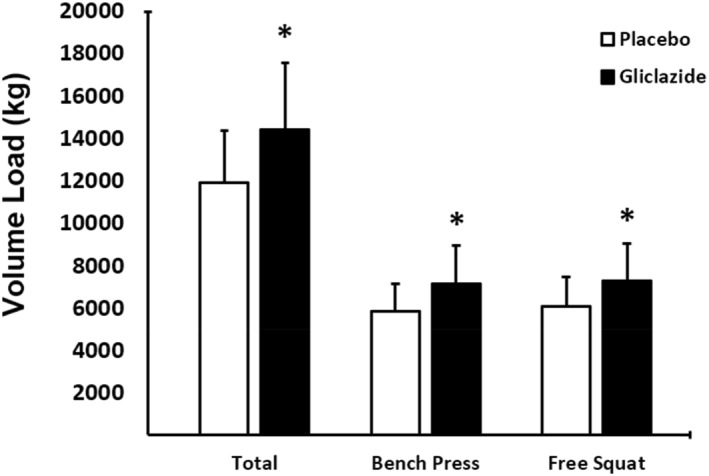
Comparison of volume‐load between the gliclazide (*n* = 44) and placebo groups (*n* = 44). Total volume‐load is the sum of values for bench press and squat exercise. Data are presented as mean ± standard deviation (SD). Differences were tested using Student's *t*‐test for paired samples for each exercise type (bench press and free squat) and for total; *p < 0.05 versus placebo.

Total VL in all strength exercise sessions (Figure 2) was greater in the gliclazide group when compared to the placebo group (*p* < 0.001; *d* = 0.88). We found similar results for both upper limb (bench press, *p* < 0.001; *d* = 0.81) and lower limb exercises (free squat, *p* < 0.001; *d* = 0.76).

Table 2 describes differences in performance by session and set. Overall, the gliclazide group showed better performance than the placebo group (Table 2). The mean difference in total VL between the gliclazide and placebo groups was 23.3% (Gliclazide: 2383.9 ± 643.3 kg; Placebo: 1721.0 ± 410.9 kg) for bench press and 23.2% for squat exercises (Gliclazide: 2470.8 ± 641.2 kg; Placebo: 1705.0 ± 428.5 kg).

**TABLE 2:** Comparison of strength exercise performance (volume‐load) by type, session and set.

	Bench press					Free squat				
	Placebo (*n* = 44)		Gliclazide (*n* = 44)			Placebo (*n* = 44)		Gliclazide (*n* = 44)		
Session 1	# reps	Load (kg)	# reps	Load (kg)	*p*‐value	# reps	Load (kg)	# reps	Load (kg)	*p*‐value
1st set	9.9 ± 1.1	805.6 ± 180.8	9.9 ± 1.2	800.1 ± 182.5	0.654	8.9 ± 1.3	807.4 ± 199.8	8.9 ± 1.5	812.4 ± 184.1	0.641
2nd set	7.4 ± 1.0	600.8 ± 143.1	8.3 ± 1.2	672.0 ± 165.2	0.001	7.4 ± 1.2	666.9 ± 166.1	7.3 ± 1.3	675.0 ± 160.8	0.204
3rd set	5.5 ± 0.9	447.3 ± 109.8	6.8 ± 1.2	550.3 ± 155.6	0.001	5.9 ± 1.0	522.3 ± 137.4	5.7 ± 1.1	535.1 ± 136.6	0.023
4th set	4.2 ± 0.9	343.1 ± 94.3	4.9 ± 1.0	400.5 ± 119.7	0.001	4.4 ± 0.8	384.6 ± 120.0	4.2 ± 1.0	398.6 ± 107.1	0.040
Total	27.1 ± 3.4	2196.8 ± 504.0	29.8 ± 4.4	2422.9 ± 609.6	0.001	26.6 ± 4.0	2381.2 ± 600.3	26.1 ± 4.5	2421.1 ± 571.0	0.042
**Session 2**	**# reps**	**Load (kg)**	**# reps**	**Load (kg)**	***p*‐value**	**# reps**	**Load (kg)**	**# reps**	**Load (kg)**	***p*‐value**
1st set	9.2 ± 0.8	744.7 ± 151.0	9.6 ± 1.3	778.8 ± 188.4	0.017	7.6 ± 1.2	684.1 ± 143.2	9.1 ± 1.2	826.8 ± 177.9	0.001
2nd set	6.3 ± 0.9	507.1 ± 123.5	7.9 ± 1.3	642.9 ± 168.1	0.001	6.2 ± 1.1	566.7 ± 138.8	7.3 ± 1.3	662.5 ± 162.5	0.001
3rd set	4.8 ± 0.8	386.3 ± 100.1	6.3 ± 1.2	510.1 ± 139.2	0.001	4.5 ± 1.0	409.0 ± 111.0	5.9 ± 1.0	534.6 ± 130.6	0.001
4th set	3.4 ± 0.8	277.7 ± 85.0	4.8 ± 1.0	390.3 ± 116.0	0.001	3.3 ± 0.9	297.0 ± 100.3	4.6 ± 0.9	420.0 ± 108.8	0.001
Total	23.6 ± 2.9	1915.8 ± 436.8	28.6 ± 4.6	2322.0 ± 598.2	0.001	21.5 ± 3.9	1956.9 ± 477.5	26.8 ± 4.0	2443.8 ± 561.3	0.001
**Session 3**	**# reps**	**Load (kg)**	**# reps**	**Load (kg)**	***p*‐value**	**# reps**	**Load (kg)**	**# reps**	**Load (kg)**	***p*‐value**
1st set	8.5 ± 1.0	689.1 ± 153.1	9.5 ± 1.7	769.7 ± 202.4	0.001	6.5 ± 1.2	592.7 ± 140.4	8.6 ± 1.8	779.8 ± 214.7	0.001
2nd set	5.4 ± 1.0	439.1 ± 113.0	8.1 ± 1.5	658.7 ± 183.5	0.001	5.3 ± 1.0	482.5 ± 113.3	7.0 ± 1.6	638.1 ± 179.8	0.001
3rd set	4.2 ± 0.7	340.5 ± 90.8	6.6 ± 1.2	534.1 ± 145.4	0.001	4.0 ± 0.9	362.2 ± 98.7	6.2 ± 1.3	566.3 ± 149.8	0.001
4th set	3.1 ± 0.7	252.3 ± 78.2	5.2 ± 1.2	421.4 ± 128.7	0.001	2.9 ± 0.9	267.6 ± 91.5	5.3 ± 0.9	486.7 ± 119.4	0.001
Total	21.2 ± 2.9	1721.0 ± 410.9	29.3 ± 5.4	2383.9 ± 643.3	0.001	18.8 ± 3.6	1705.0 ± 428.5	27.2 ± 5.3	2470.8 ± 641.2	0.001

*Note:* The values indicate total volume‐load (VL) calculated by multiplying VL in kg (80% 1‐RM) x number of repetitions per set. 1‐RM: one‐repetition maximum. Data are presented as mean ± standard deviation (SD). Differences were tested using Student's *t*‐test for paired samples for each exercise type (bench press and free squat) and considered significant at *p* < 0.05.

### 3.2 Muscle Strength Recovery

**FIGURE 3:**
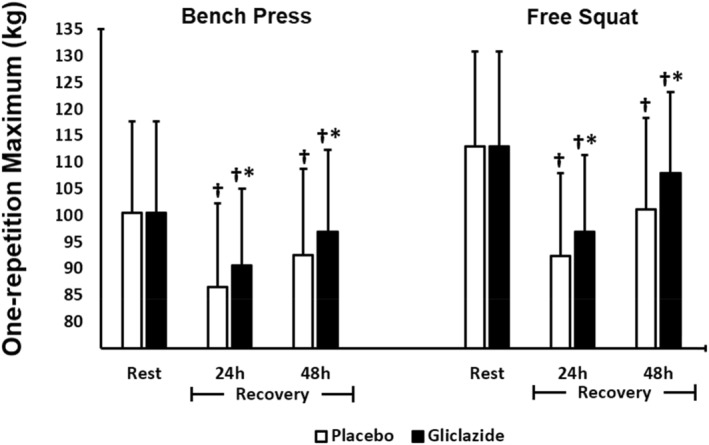
Comparison of one‐repetition maximum (1‐RM) between the gliclazide (*n* = 44) and placebo groups (*n* = 44). Data are presented as mean ± standard deviation (SD). Differences were tested by two‐way ANOVA with repeated measures by exercise type (bench press and free squat) and time and Bonferroni post‐hoc test; *p < 0.05 versus placebo; † *p* < 0.05 versus baseline within the group.

1‐RM measures were lower 24 and 48 h after exercise during recovery compared to baseline (pre‐intervention). However, recovery was more accelerated in the gliclazide group in all comparisons (exercise x time) for bench press (24h: *p* < 0.001, *d* = 0.26; 48h: *p* < 0.001, *d* = 0.28) and free squat (24h: *p* < 0.001, *d* = 0.30; 48h: *p* < 0.001, *d* = 0.41). These data show that gliclazide improved recovery of muscle strength (Figure 3).

**FIGURE 4:**
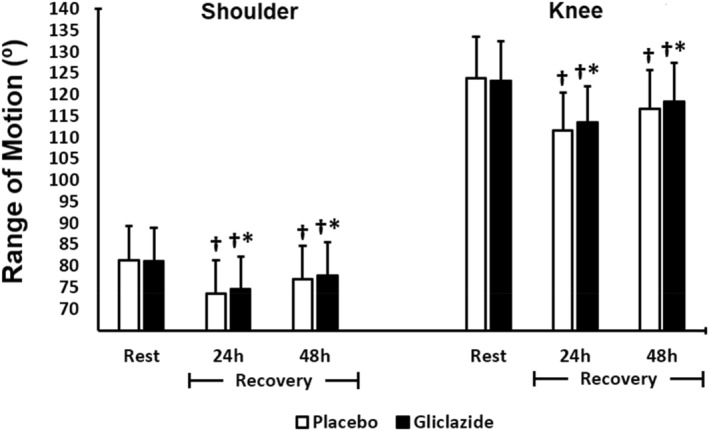
Comparison of range of motion (ROM) between the gliclazide (*n* = 44) and placebo groups (*n* = 44). Data are presented as mean ± standard deviation (SD). Differences were tested by two‐way ANOVA with repeated measures for paired samples by joint (shoulder and knee) and time and Bonferroni post‐hoc test; *p < 0.05 versus placebo; † *p* < 0.05 versus baseline within the group.

Similar to 1‐RM results, shoulder and knee ROM measures were lower 24 and 48 h of recovery when compared to baseline (pre‐intervention) (Figure 4). However, the gliclazide group showed faster recovery compared to placebo 24 h (knee ROM *p* < 0.001, *d* = 0.22; shoulder ROM *p* = 0.004, *d* = 0.13) and 48 h after the last exercise session (knee ROM *p* < 0.001, *d* = 0.20, shoulder ROM *p* = 0.048, *d* = 0.09) (Figure 4).

**FIGURE 5:**
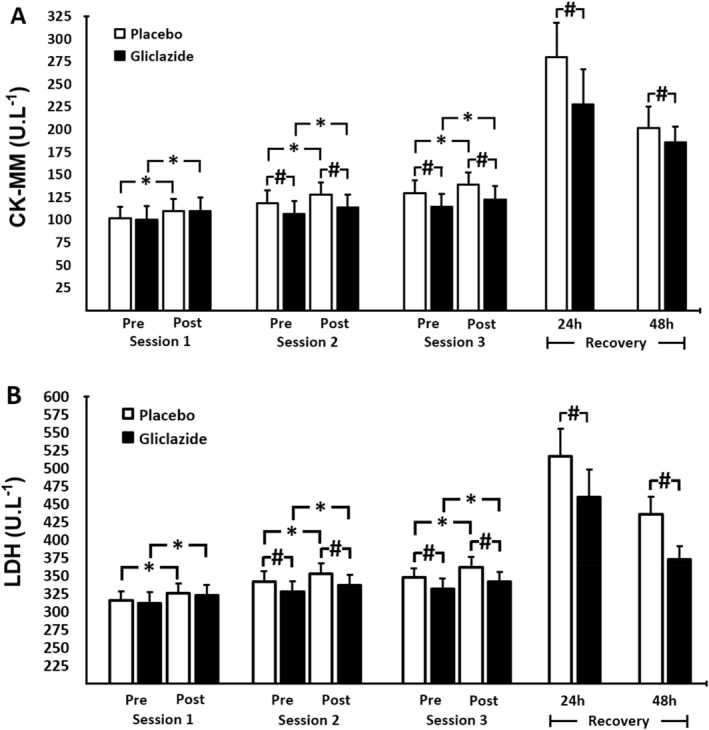
Comparison of muscle damage markers between the gliclazide (*n* = 44) and the placebo groups (*n* = 44). Panel A: enzymatic activity of creatine kinase MM isoform (CK‐MM). Panel B: serum levels of lactate dehydrogenase (LDH). Data are presented as mean ± standard deviation (SD). Differences were tested by two‐way ANOVA with repeated measures and Bonferroni post‐hoc test; ^#^ *p* < 0.05 versus placebo at the same time and * *p* < 0.05 versus pre‐session within the group.

The activity of CK‐MM increased immediately post‐exercise session in response to strength exercise in both the gliclazide and placebo groups in within‐group comparisons (*p* < 0.001 for all comparisons) (Figure 5A). Starting from session 2, and considering the cumulative effect of strength exercise (pre‐ and post‐session assessments), CK‐MM was consistently lower in the gliclazide group compared to placebo. The same pattern of response was seen 24 and 48 h of recovery with CK‐MM being lower in the gliclazide group. Interestingly, LDH levels were also consistently lower in the gliclazide group (Figure 5B). As expected, our results point to intentional muscle damage in response to the exercise protocol and show that the use of gliclazide MR appears to be associated with enhanced muscle recovery despite the cumulative effect of exercise sessions.

**FIGURE 6:**
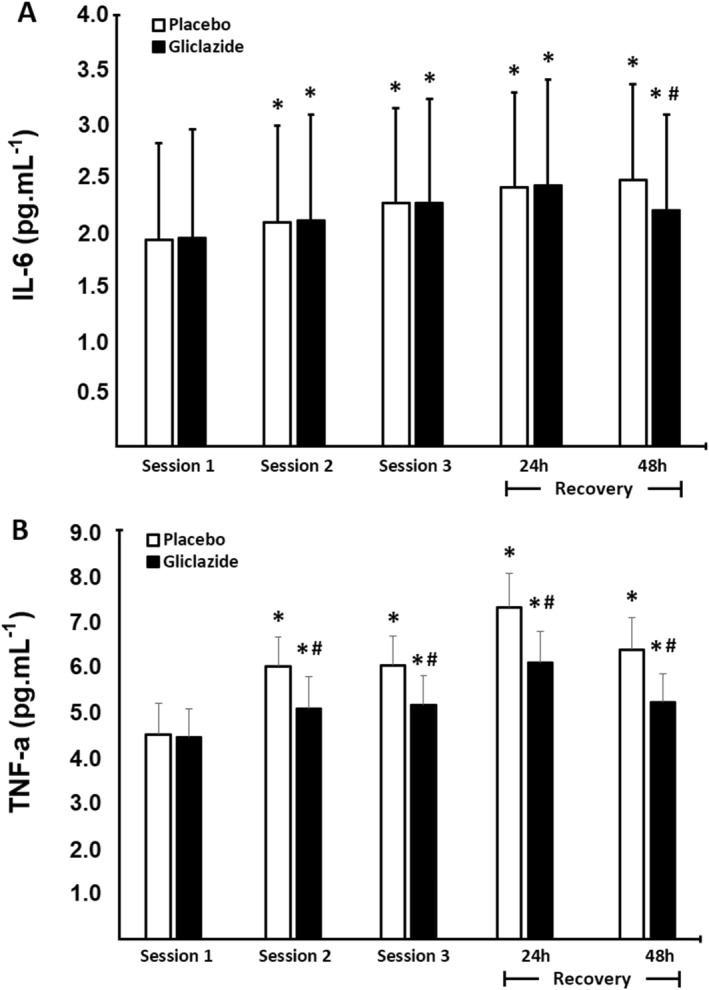
Comparison of inflammatory markers between the gliclazide (*n* = 44) and the placebo groups (*n* = 44). Panel A: interleukin 6 (IL‐6). Panel B: tumor necrosis factor alpha (TNF‐α). Data are presented as mean ± standard deviation (SD) pre‐session and 24 and 48 h of recovery. Differences were tested by Generalized Estimating Equations and Bonferroni post‐hoc test; *p < 0.05 versus session 1 within the group; ^#^ *p* < 0.05 versus placebo at the same time.

Inflammatory markers were measured before exercise sessions and 24 and 48 h of recovery (Figure 6A). At baseline, no difference between groups (*p* = 0.687) was observed. Later, IL‐6 levels increased successively over exercise sessions as well as 24 and 48 h of recovery (*p* < 0.001 for all comparisons) when compared to baseline. In addition, we found no differences between the groups, except 48 h after exercise where IL‐6 levels were lower in the gliclazide group compared to placebo (*p* < 0.001, *d* = 0.31). In turn, TNF‐α levels increased continuously from baseline in both groups (*p* < 0.001 for all comparisons) (Figure 6B). However, TNF‐α levels were lower in the gliclazide group compared to placebo in all times evaluated as follows: session 1 (baseline), *p* = 0.663; session 2, *p* < 0.001 and *d* = 1.37; session 3, *p* < 0.001 and *d* = 1.33; 24 h of recovery, *p* < 0.001 and *d* = 1.70; and 48 h of recovery, *p* < 0.001 and *d* = 1.70 (Figure 6B).

Regarding muscle soreness intensity scores, from session 2, mean muscle soreness ratings were lower in the gliclazide group than placebo (Supporting Information S1: Table S1). Overall, during the recovery period (24 and 48 h post‐exercise), muscle soreness ratings decreased by 38% in both groups though they were lower in the gliclazide group compared to placebo (Supporting Information S1: Table S1). Finally, compared to placebo, the gliclazide group showed higher scores of perceived recovery pre‐exercise in all sessions and during recovery (Supporting Information S1: Table S1).

By contrast, hemodynamic measures—heart rate (HR), blood pressure (BP), and rate–pressure product (double product)—did not change significantly before, during, or after the strength‐training sessions (Supporting Information S1: Table S2).

### 3.3 Insulin and Glucose Levels and Adverse Events

As expected, plasma insulin concentrations were consistently higher in the gliclazide condition compared with placebo across all assessed time points; *p* = 0.005. Before each exercise session, insulin levels were greater in the gliclazide group (Session 1: 7.8 ± 1.2 vs. 5.7 ± 0.8 µU·mL^−1^; Session 2: 7.9 ± 1.2 vs. 5.7 ± 0.9 µU·mL^−1^; Session 3: 7.9 ± 1.0 vs. 5.7 ± 0.8 µU·mL^−1^; *p* < 0.001 for all comparisons). Similarly, during the recovery period, insulin concentrations remained higher with gliclazide at 24 h (8.0 ± 0.9 vs. 5.8 ± 0.8 µU·mL^−1^) and 48 h (8.0 ± 0.9 vs. 5.9 ± 0.8 µU·mL^−1^; *p* < 0.001 for all comparisons).

Also, we found an acute reduction in blood glucose levels in all exercise sessions in both groups (Table 3). However, the magnitude of reduction was greater in the gliclazide group: in session 1, blood glucose reduced by 23.1% in the gliclazide group compared to 9.3% with placebo (*p* < 0.001; *d* = 1.32), in session 2, it reduced by 23.5% versus 11.8% (*p* < 0.001; *d* = 1.09), and in session 3, 24.8% versus 9.2% (*p* < 0.001; *d* = 1.21).

**TABLE 3:** Comparison of plasma glucose levels before and after the exercise sessions.

	Placebo (*n* = 44)				Gliclazide (*n* = 44)			
	Pre‐exercise (mg/dL)	Post‐exercise (mg/dL)	Δ (mg/dL)	*p*‐value	Pre‐exercise (mg/dL)	Post‐exercise (mg/dL)	Δ (mg/dL)	*p*‐value (post‐hoc)
Session 1	115.0 ± 18.5	104.3 ± 15.3	10.6 ± 16.7	0.034	113.1 ± 9.5	86.9 ± 10.2	26.2 ± 8.8	< 0.001
Session 2	115.2 ± 17.3	106.1 ± 16.5	13.8 ± 15.2	0.039	114.8 ± 9.5	87.8 ± 11.4	27.0 ± 8.5	< 0.001
Session 3	117.1 ± 15.8	105.3 ± 14.4	11.8 ± 16.8	0.019	116.6 ± 9.8	87.6 ± 13.4	28.9 ± 10.4	< 0.001

*Note:* Pre‐exercise, immediately before exercise session; post‐exercise, immediately after the exercise session. Data are presented as mean ± standard deviation (SD). Differences were tested using two‐way ANOVA with Bonferroni post‐hoc test and considered significant at *p* < 0.05; *p* (placebo/gliclazide vs. Δ—change in each session) < 0.001.

Three participants in the gliclazide group (7%) experienced adverse hypoglycemic events post‐exercise (blood glucose levels of 46, 49 and 51 mg/dL) as dizziness, pale skin and sweating were common signs. This effect was reversed immediately with the consumption of 30g of quickly absorbed carbohydrate—maltodextrin (Integralmedica) containing ∼27g carbohydrates.

## 4 Discussion

To the best of our knowledge, this is the first study that primarily aimed to examine whether the use of gliclazide MR 90 mg enhances performance and/or muscle recovery in strength‐trained healthy volunteers following three strength exercise sessions. Based on our main findings, we accept the hypothesis that gliclazide MR 90 mg acutely enhances strength performance (assessed by VL) and muscle recovery (assessed by CK‐MM, LDH, TNF‐α and IL‐6 levels and 1‐RM and ROM recovery) and improves perceived muscle soreness and recovery. However, the use of gliclazide MR at a dose of 90 mg can cause adverse effects, especially associated with reduced blood glucose levels leading to hypoglycemic events.

The rationale for using gliclazide in amateur and professional athletes is that it stimulates endogenous insulin secretion (Draznin 2022), thereby optimizing nutrient uptake—primarily glucose and amino acids—into skeletal muscle (Flores‐Opazo et al. 2020; Richter et al. 2021). Greater intramuscular glucose availability and accelerated glycogen resynthesis (van Loon et al. 2000) support ATP production for moderate‐to‐high–intensity efforts (Mata et al. 2019). In parallel, increased amino‐acid availability augments the stimulus for muscle protein synthesis, which can enhance recovery (Ivy 2004). Both processes—glycogen resynthesis and protein synthesis—are insulin‐facilitated (Sylow et al. 2021) and, in our hypothesis, potentiated by gliclazide.

Change in exercise capacity has been used as a parameter of strength performance (Correa et al. 2013; Correa 2012). We tested a high‐intensity exercise protocol (80% of 1‐RM) (ACSM 2009; NSCA 2021). There is a consensus in the literature that moderate‐high intensity exercise performance is dependent on greater availability of nutrients especially carbohydrate in the form of glucose/glycogen stores (Hargreaves and Spriet 2020). During exercise, the secretion of endogenous insulin is reduced. Insulin‐independent mechanisms promote glucose uptake by muscle cells in response to muscle contraction which is possibly a compensatory effect to reduced insulin levels during exercise (Richter et al. 2021). The insulinotropic action of gliclazide is not a response to exercise. So, there is “twice the effect” on glucose uptake in muscle cells during exercise: through insulin‐independent mechanisms triggered by muscle contraction and increased circulating levels of insulin due to pharmacological stimulation. This could explain enhanced performance seen as early as the first exercise session of our study. In contrast, we reported previously that a single‐dose administration of gliclazide MR 60 mg did not improve performance in a single session of strength exercise at 65% of 1‐RM (unpublished data). The ergogenic effect of this drug may be dose‐dependent, requiring a higher dose and may involve exercise at greater intensity to elicit a metabolic response.

The assumption of increased glucose uptake during strength exercise is further supported by the fact that three participants in the gliclazide group experienced hypoglycemic events during exercise. In addition, the participants in the placebo group probably experienced residual fatigue as a result of consecutive exercise sessions as their performance dropped by 8% from session 1 to session 2 and by 11.5% from session 2 to session 3. This decline in performance was not seen in the gliclazide group (Figure 2). It could be that more rapid muscle glycogen restoration occurred between sessions in the gliclazide group, though we did not measure glycogen in the present study.

We evaluated 1‐RM and ROM as functional parameters of muscle recovery. Exercise‐induced muscle damage has been associated with the ability to generate maximum dynamic strength following an exercise session (Morton et al. 2005). In our study, at 24 h post‐exercise, maximal strength—assessed by one‐repetition maximum (1‐RM)—decreased by 9.8% (bench press) and 13.3% (squat) in the gliclazide group, compared with 16.6% and 18.6%, respectively, in the placebo group. Then the participants recovered 96.1% (bench press) and 95.6% (squat) of their strength in the gliclazide group and 92.1% (bench press) and 90.3% (squat) in the placebo Group 48 h post‐exercise. Though these values are small, they may indicate potential ergogenic effects of gliclazide on recovery of muscle strength. Similar effects have been shown for other interventions, such as nutritional supplements ingestion such as creatine, carbohydrates and proteins (Isenmann et al. 2019; Lanhers et al. 2015). We did not find in the literature data of these effects with the use of gliclazide. In turn, the ROM for shoulder horizontal abduction and knee extension is another parameter of muscle recovery (Brentano et al. 2017; Romero‐Moraleda et al. 2019) assessed in this study. Our findings were similar to those for 1‐RM. Reduced ROM seen in the placebo group during recovery (24 and 48 h post‐exercise) is consistent with that reported for control groups in other studies testing nutritional interventions (Isenmann et al. 2019; Lanhers et al. 2015). Mean ROM reduction was 9% with nearly 95% recovery 48 h after exercise. It suggests that our sample was comparable to those used in other studies, which further supports our findings of the effects of gliclazide MR on muscle recovery.

Indirect markers of exercise‐induced muscle damage, mainly CK‐MM and LDH, are widely used to quantitatively assess the effects of exercise on muscle recovery (Koch et al. 2014; Romero‐Moraleda et al. 2019). In the present study, CK‐MM and LDH exhibited similar response patterns, consistent with previous findings (Bernat‐Adell et al. 2021). The exercise protocol induced acute increases in both markers across sessions, reflecting the expected response to high‐intensity resistance exercise. From the second session onward, pre‐exercise CK‐MM and LDH levels were lower in the gliclazide group compared with placebo, suggesting improved recovery from muscle damage induced by previous sessions. Peak CK‐MM and LDH concentrations were observed after the final training session, a finding that is in line with other studies using resistance exercise protocols (Brentano et al. 2017; Doma et al. 2022), although peak timing may vary depending on the exercise design (Machado et al. 2011). With respect to CK‐MM specifically, peak concentrations may occur between 24 and 72 h after exercise, particularly following unaccustomed or eccentric loading (Brancaccio et al. 2010; Clarkson and Hubal 2002). In the present study, a repeated high‐intensity resistance exercise protocol was employed, consisting of three sessions performed 48 h apart, which modifies the classic single‐bout CK time course. In such repeated‐bout designs, attenuated CK elevations before subsequent sessions are commonly attributed to the repeated‐bout effect, whereas peak CK concentrations may emerge after the final session as a consequence of cumulative mechanical and metabolic stress (Doma et al. 2022; Machado et al. 2012). Accordingly, the elevated CK observed after the third training session likely reflects the summative impact of repeated loading rather than an inconsistency in CK kinetics. Importantly, comparisons between conditions consistently showed lower CK‐MM activity in the gliclazide group across exercise sessions and recovery, supporting an accelerated recovery response despite the cumulative exercise stimulus. This effect may be partly explained by enhanced glucose uptake by myocytes resulting from increased endogenous insulin secretion induced by gliclazide, leading to greater cellular energy availability and potentially facilitating repair processes, as well as increased intracellular water content via osmotic mechanisms.

Also, changes in IL‐6 and TNF‐α concentration levels are often used as markers of the inflammatory response (Donges et al. 2010; Ebbeling and Clarkson 1989). The mechanical overload of concentric and eccentric contractions during strength exercises primarily induces functional adaptations in skeletal muscles due to transient desired structural changes in sarcomeres. During recovery, exercise‐induced damage results in muscle inflammation and, consequently, sarcomere repair (Ebbeling and Clarkson 1989). Here, the protocol of strength exercises caused a continuous increase in IL‐6 levels across all sessions, as well as during recovery 24 h after exercise. These findings corroborate that reported in other studies (Febbraio and Pedersen 2005; Serrano et al. 2008) suggesting that IL‐6 levels increase in response to metabolic and mechanical stress that occur with exercise. Bartolomei study (Bartolomei et al. 2017) showed that, after a single session of strength exercise at high intensity or with high VL, IL‐6 concentration returned to pre‐exercise levels 24 and 48 h of recovery. Interestingly, muscle damage markers (CK‐MM and LDH) showed similar response in our study. IL‐6 levels were increased in the placebo group and reduced in the gliclazide Group 48 h of recovery. This finding suggests that gliclazide has an ergogenic effect, accelerating muscle recovery and reducing inflammation. We also evaluated plasma TNF‐α concentration, which clearly increased across the three exercise sessions of the study and then it remained high over a 24‐h period. Considering that IL‐6 and TNF‐α were essentially pro‐inflammatory cytokines, this finding reinforces their action to reduce exercise‐induced inflammation.

Several mechanisms may underlie the anti‐inflammatory effects of gliclazide. Clinically, treatment with modified‐release gliclazide in type 2 diabetes reduces plasma IL‐6 with a concomitant trend toward lower TNF‐α, suggesting actions beyond glycemic control (Drzewoski and Zurawska‐Klis 2006). At the cellular level, gliclazide inhibits the AGE–RAGE/TLR4 signaling cascade, attenuates reactive oxygen species generation, and suppresses NF‐κB activation, thereby decreasing pro‐inflammatory cytokine expression; in parallel, it promotes macrophage polarization toward the anti‐inflammatory M2 phenotype (Jahan and Choudhary 2021). Additionally, as an insulin secretagogue, gliclazide enhances glucose uptake into skeletal muscle, potentially improving substrate availability and contributing to tissue repair.

Thus, reduced cytokine release (IL‐6, TNF‐α) and improved nutrient delivery to muscle (glucose and amino acids) would be expected to lower oxidative stress and limit sarcolemmal disruption during recovery. Consequently, less intracellular content (CK‐MM, LDH) leaks into the circulation, yielding lower plasma concentrations at 24–48 h post‐exercise—a biochemical profile consistent with attenuated residual muscle damage and a more efficient repair process. Accordingly, markers of muscle damage and post‐exercise recovery (CK‐MM, LDH, IL‐6, and TNF‐α) were lower in the gliclazide group than in the placebo group, and perceived muscle soreness and recovery scores were also improved with gliclazide, further supporting enhanced recovery after strength exercise. Importantly, muscle pain assessed during exercise sessions reflects acute effort‐related muscle discomfort rather than delayed‐onset muscle soreness (DOMS). These pain constructs involve distinct physiological mechanisms and should not be interpreted interchangeably.

Our study has some limitations. The main limitation lies in the unfeasibility to evaluate the effects of gliclazide for longer periods of time, which is related to ethical aspects. A second limitation is that only plasma glucose levels, but not insulin, were measured at different times. Concurrent plasma glucose and insulin measurements would have allowed a more in‐depth discussion of their association with exercise. Another important point is that we did not examine counter‐regulatory effects of anti‐inflammatory cytokines on IL‐6 and TNF‐α. Still, we believe that these limitations do not weaken our results.

In conclusion, this randomized, placebo‐controlled, double‐blind crossover randomized clinical trial showed that gliclazide MR 90 mg at single daily dose taken 8 h before strength exercise sessions had an ergogenic effect, enhancing strength performance and accelerating muscle recovery. However, these benefits were counterbalanced by clinically relevant glucose reductions, including three symptomatic hypoglycemic episodes. Any consideration of off‐label use in athletic contexts must therefore weigh potential performance benefits against safety risks.

## Author Contributions

J.B.M. and A.M.L. designed the study. J.B.M. collected and analyzed the data. J.B.M., T.D., B.D.S. and A.M.L. carried out the interpretation of data. J.B.M. drafted the manuscript. T.D., B.D.S and A.M.L. helped prepare the manuscript and revised it critically. All authors contributed to manuscript preparation, reviewed and approved its final version.

## Funding

The authors have nothing to report.

## Ethics Statement

The study was approved by the research ethics committee at Instituto de Cardiologia do Rio Grande do Sul/Fundação Universitária de Cardiologia (#3771137). All volunteers signed a free informed consent form prior to entering the study.

## Conflicts of Interest

The authors declare no conflicts of interest.

## Supporting information


Supporting Information S1

## Data Availability

Availability of data and material: The dataset supporting the conclusions of this article is included within the article and its additional file.
