# Supplementary material for: Gliclazide Enhances Exercise Performance and Recovery of Muscle Strength in Healthy Trained Individuals: A Randomized Controlled Trial
Source: Eur J Sport Sci. 2026 Mar 12;26(4):e70155. doi: 10.1002/ejsc.70155 (PMC13140689; doi:10.1002/ejsc.70155)
Supplement: Supplementary file 1 — Supporting Information S1 [file EJSC-26-e70155-s001.docx]

**Supplementary Material**

| **Table S1 –** Comparison of perceived subjective variables between the study groups | | | |
| --- | --- | --- | --- |
|  | **Placebo (n = 44)** | **Gliclazide (n = 44)** | **p-value** |
| Perceived muscle soreness scores | |  |  |
| Session 1 | 3.5 ± 0.5 | 3.4 ± 0.7 | 0.094 |
| Session 2 | 4.3 ± 0.6 | 4.0 ± 0.5 | <0.001 |
| Session 3 | 4.8 ± 0.6 | 4.2 ± 0.5 | <0.001 |
| Post-24h | 4.3 ± 0.9 | 3.2 ± 0.9 | <0.001 |
| Post-48h | 2.7 ± 1.0 | 2.0 ± 0.9 | <0.001 |
| Perceived recovery scores |  |  |  |
| Session 1 | 10.0 ± 0.01 | 10.0 ± 0.01 | 1.000 |
| Session 2 | 2.8 ± 0.7 | 3.8 ± 0.7 | <0.001 |
| Session 3 | 2.8 ± 0.7 | 3.8 ± 0.7 | <0.001 |
| Post-24h | 2.8 ± 0.9 | 3.8 ± 0.9 | <0.001 |
| Post-48h | 4.8 ± 1.1 | 5.9 ± 1.1 | <0.001 |

Post-24h, 24 hours of recovery; post-48h, 48 hours of recovery. Data are presented as mean ± standard deviation (SD) regarding pre-sessions, and 24 hours and 48 hours of recovery. Differences were tested using Student’s t-test for paired samples for each session and recovery time, and considered significant at p<0.05.

| **Table S2 –** Comparison of hemodynamic variables measured in the study | | | |
| --- | --- | --- | --- |
|  | **Placebo (n = 44)** | **Gliclazide (n = 44)** | **p-value** |
| HR (bpm) |  |  |  |
| Session 1 | 153.0 ± 15.5 | 151.4 ± 14.7 | 0.644 |
| Session 2 | 154.6 ± 16.6 | 156.4 ± 13.9 | 0.610 |
| Session 3 | 157.3 ± 14.6 | 152.6 ± 15.8 | 0.196 |
| Post-24h | 77.3 ± 7.0 | 75.4 ± 6.3 | 0.204 |
| Post-48h | 75.4 ± 7.8 | 72.5 ± 5.6 | 0.059 |
| SBP (mmHg) |  |  |  |
| Session 1 | 145.4 ± 14.6 | 148.3 ± 13.3 | 0.324 |
| Session 2 | 147.6 ± 13.7 | 145.7 ± 12.3 | 0.471 |
| Session 3 | 148.6 ± 15.2 | 146.2 ± 14.2 | 0.431 |
| Post-24h | 116.5 ± 7.6 | 115.2 ± 8.5 | 0.450 |
| Post-48h | 118.5 ± 9.8 | 117.6 ± 8.9 | 0.664 |
| DBP (mmHg) |  |  |  |
| Session 1 | 75.7 ± 9.4 | 74.1 ± 8.4 | 0.376 |
| Session 2 | 76.3 ± 8.3 | 77.6 ± 7.5 | 0.425 |
| Session 3 | 78.5 ± 7.7 | 76.7 ± 9.1 | 0.303 |
| Post-24h | 69.6 ± 5.9 | 70.6 ± 6.8 | 0.447 |
| Post-48h | 70.4 ± 6.1 | 72.5 ± 6.6 | 0.130 |
| Double product |  |  |  |
| Session 1 | 22,212.0 ± 2,865.2 | 22,461.5 ± 2,962.7 | 0.676 |
| Session 2 | 22,762.3 ± 2,722.1 | 22,785.4 ± 2,722.6 | 0.967 |
| Session 3 | 23,338.0 ± 2,892.7 | 22,331.4 ± 3,249.6 | 0.132 |
| Post-24h | 9,010.4 ± 1,1226.2 | 8,694.4 ± 1013.9 | 0.159 |
| Post-48h | 8,945.1 ± 1,218.1 | 8,525.0 ± 848.0 | 0.064 |

HR, heart rate; SBP, systolic blood pressure; DBP, diastolic blood pressure; bpm, beats per minute; post-24h, 24 hours of recovery; post-48h, 48 hours of recovery. Differences were tested using Student’s t-test for paired samples for each session and recovery time, and considered significant at p<0.05.
